# A New Mission Awaits: Machine Learning to Predict COVID-19–Related Complications∗

**DOI:** 10.1016/j.jacadv.2022.100045

**Published:** 2022-05-08

**Authors:** Shaobo Shi, Qiqi Cao

**Affiliations:** a Department of Cardiology, Renmin Hospital of Wuhan University, Wuhan, China; b Cardiovascular Research Institute, Wuhan University, Wuhan, China; c Hubei Key Laboratory of Cardiology, Wuhan University, Wuhan, China

**Keywords:** cardiovascular complications, COVID-19, machine learning

**Figure:**
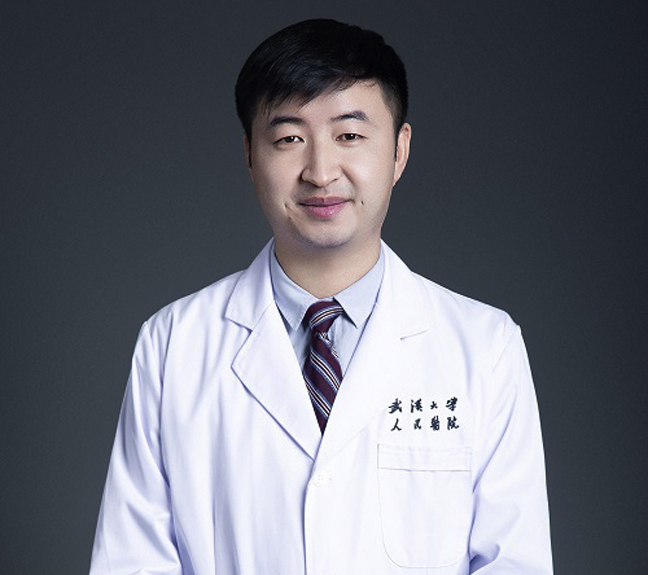

The COVID-19 pandemic is still raging around the world, infecting more than 500 million people and claiming more than 6.2 million lives.^1^ Serious cardiovascular complications are a major cause of death in hospitalized patients with COVID-19, and to reduce morbidity and mortality, prediction of cardiovascular events and prompt treatment is required. In this issue of *JACC: Advances*, Shade et al^2^ apply machine learning to develop a real-time predictive tool to assess all-cause mortality/cardiac arrest (AM/CA) and thromboembolic events (TEs), a tool which has significant clinical potential to improve management and reduce emergencies in patients with COVID-19.

Although COVID-19 is a respiratory infection caused by the severe acute respiratory syndrome-coronavirus-2 (SARS-CoV-2), it can target the heart and blood vessels and result in cardiovascular complications. During the early stage of the epidemic, myocardial damage caused by SARS-CoV-2 infection was very common. Shi et al^3^ found that myocardial damage occurred in as many as 19.7% of patients with COVID-19 and was associated with a 4.26-fold increased risk of death. Furthermore, COVID-19 patients who have cardiovascular disease are a high-risk group with poor prognosis, and the majority of deaths are attributable to new-onset cardiovascular complications or deterioration in cardiac status, including adverse events such as cardiac arrests, acute heart failure, acute myocardial infarction, venous thrombosis, acute myocarditis, and even stress cardiomyopathy. Currently, infection with the Omicron virus is rampant, with rapid spread and a large number of infected people. It is said that no one is spared. An important task facing the government and society is to identify high-risk patients and prevent morbidity and mortality; however, this remains a very difficult problem and the solutions require expertise from medicine, biology, engineering, informatics, and other related disciplines. Teams must work together to create new tools, new drugs, and new solutions to solve this urgent problem.

Risk stratification is crucially important for the hospitalized patient with COVID-19 so that treatment strategies can be planned to minimize morbidity and mortality. Previous studies on risk stratification have used traditional and interpretable modeling methods to predict mortality.^4^ In the current study, Shade et al.^2^ apply machine learning techniques to create a real-time tool, named COVID-HEART, to predict adverse events (AM/CA and TE) in COVID-19 patients and to provide a continuously evolving warning system for impending events. COVID-HEART was derived from routinely acquired clinical parameters and biomarkers and achieved excellent predicting capability for the 2 outcomes (AM/CA and TE), with better performance in predicting AM/CA than TE. In practice, COVID-HEART comprehensively assesses the combined effects of many variables and provides a novel and accurate risk score.

Despite the encouraging findings, several issues need to be considered when developing and using these types of clinical tools. The COVID-HEART model used real-world data, and the importance of raw data cleanliness is very important for any machine learning model. Although many types of clinical data were included, additional variables may be important. For instance, the type of COVID-19 variant may be important because the biological characteristics of each SARS-CoV-2 variant differ and the clinical presentations are heterogeneous.^5^ Whether the results of the existing training are universal is debated. This concern can be addressed if the tools have strong and stable self-learning ability. Finally, in addition to predicting mortality and thrombosis, other cardiovascular complications may be equally as important and will require additional prediction models. All clinical risk predictions tools have advantages and disadvantages, and it is important to understand shortcomings when using models/tools and artificial intelligence tools are no exception.

Urgent clinical needs have resulted in new solutions and have also provided an opportunity for the use of new technologies, such as machine learning, to demonstrate their utility. Strictly validated, practical, stable, and reliable tools will help to improve clinical care and reduce cardiovascular complications.

## Funding support and author disclosures

This research was supported by Grants from the Nature Science Foundation of China (No: 81800447), and the Nature Science Foundation of Hubei province (No: 2017CFB204). The authors have reported that they have no relationships relevant to the contents of this paper to disclose.
